# On the structure of nanoparticle clusters: effects of long-range interactions†

**DOI:** 10.1039/d4cp04235b

**Published:** 2025-01-21

**Authors:** Rens Kamphorst, Maximilian F. Theisen, Ankur D. Bordoloi, Samir Salameh, Gabrie M. H. Meesters, J. Ruud van Ommen

**Affiliations:** a Department of Chemical Engineering, Delft University of Technology Van der Maasweg 9 Delft 2629HZ The Netherlands R.Kamphorst@tudelft.nl J.R.vanOmmen@tudelft.nl; b FH Münster Münster Germany

## Abstract

The fractal structure of aggregates consisting of primary nanoparticles naturally arises during their synthesis. While typically considered to be a fully stochastic process, we suspect long-range interactions, in particular van der Waals forces, to induce an active pull on particles, altering the clustering process. Using an off-grid 3D model, we show that an active pull decreases the density and fractal dimension of formed clusters. These findings could not be reproduced by 2D models, which underestimate screening effects. Additionally, we determined the range within which van der Waals forces dominate the aggregation process.

## Abstract 2

An active pull on particles undergoing Brownian motion in particle-cluster aggregation leads to more open structures. Aggregates formed under these conditions are shown to have lower fractal dimensions and densities.

## Graphical abstract

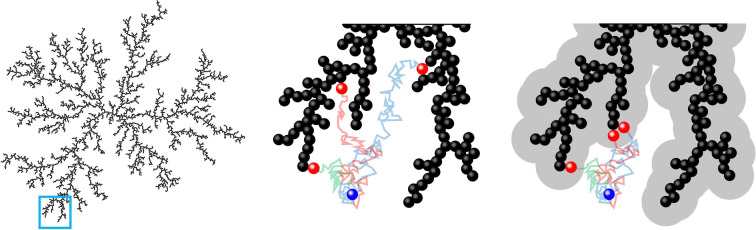

## 1. Introduction

The famous artworks in the Chauvet Cave, drawn in charcoal, suggest intentional material production by pyrolysis dating back to at least 30 000 BCE, in the Upper Paleolithic. The first evidence of systematic particle production by pyrolysis appears around 1500 BCE with the manufacture of carbon black, which was used as a pigment.^1^ However unintentional, this also marks the first large scale production of nanoparticles. Driven by the increasing interest in nanoparticles, the field has flourished over the past decades, expanding its repertoire to include a diverse array of materials with versatile applications, including photoelectric devices,^2,3^ construction materials^4–6^ and medicine.^7,8^ Most of these applications rely on the high specific surface area and low density of nanoparticle structures.

During the production of nanoparticles by pyrolysis, a precursor liquid is atomized at a high temperature, forming nano-sized droplets, which solidify upon cooling down, forming the primary nanoparticles.^9^ In a typical modern aerosol-based nanoparticle manufacturing setup pyrolysis is performed in a flame reactor, usually enriched with oxygen, methane or hydrogen.^10,11^ The macro-properties, such as bulk density and surface area, of produced nanopowders arise from their multi-scale structure. While in the flame, exact droplet size is subject to process parameters, but is typically found in the range of 5–50 nm.^12^ Outside of the flame, the temperature decreases, halting coalescence and making aggregation by sintering, where primary particles stick together due to surface diffusion, the dominant growth mechanism. When cooled to beyond the point where sintering occurs, agglomeration, induced by van der Waals forces, takes over as the main growth mechanism. The initially formed agglomerates are often referred to as ‘simple agglomerates’, as, upon further handling, they themselves can form even larger clusters called ‘complex agglomerates’.^13,14^ Aggregate sizes typically span from 100 nm to 1 μm, whereas simple agglomerate sizes range from 1–100 μm.^13–15^ While long-range interactions may contribute to the formation of larger structures too, in this work we exclusively consider the formation of aggregates.

The study of aggregate formation typically focuses on the emergence of fractal structures, that influence macro-scale properties like density and surface area. These structures arise from screening effects in the entropic process of cluster formation by Brownian droplets and particles.^16,17^ In addition to screening, the fractal dimension and eventual density of the formed aggregate may be subject to long and short-range interactions, in particular the van der Waals force. In the context of modelling the effect of attractive forces, the interactions can be represented by a capture distance, *ℓ*_c_. This denotes the critical distance between two particles at which moving any closer leads to an inevitable collision induced by attractive inter-particle forces scaling with distance. While the existence of such a zone is not established in relation to the clustering of nanoparticles, we deem its presence to be likely. There is a limited body of work that covers the potential effect of this region on the cluster structure. The classic work of Meakin^18^ and a recent study by Nicolás-Carlock *et al.*,^19^ explored the impact of these interactions on the fractality of formed structures. Both studies used 2D random walk particle-cluster models with a variable capture distance to represent attractive forces. These works conclude that fractal dimension of clusters produced with and without capture effects end up having the same fractal dimension, provided the clusters are large enough. However, while not elaborated on in those works, cluster density is highly dependent on capture distance. This is easy to overlook as clusters with the same fractal dimension can still have vastly different densities, as fractal dimension relates to the change in density with respect to cluster size, not the absolute density itself. From the prior studies it can therefore already be inferred that long-range forces have the potential to affect macro properties, even while the fractality remains unchanged. Nevertheless, whether the magnitude of the forces acting on Brownian droplets and particles within flame pyrolysis is sufficient to induce any change in particle properties remains unexplored. Furthermore, it is unclear whether large enough clusters are actually attained during aggregate formation to reach a size at which fractal dimensions converge. Given the geometric nature of screening effects, examining their impact on structure formation in 2D may be an oversimplification. Given the small size of primary nanoparticles, we hypothesize an active capture zone to be present, which should in turn affect the macroproperties of formed aggregates.

In this work, we set out to determine the van der Waals-induced capture distance for Brownian nanoparticles. Additionally, using a model, we show the effect of capture effects on fractal dimensions and densities of 3D clusters.

Our results may also have implications for other systems of clustering particles subjected to long-range forces. For instance, magnetic nanoparticles tend to aggregate, causing issues in their application for magnetic resonance imaging.^20^ Furthermore, in colloidal and biological systems exact interactions are highly dependent on surface groups and the nature of the surrounding medium,^21,22^ but are known to act on distances exceeding 1 nm.^23^

## 2. Methods

**Fig. 1:**
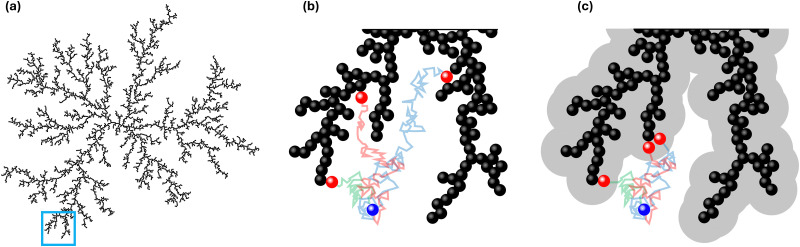
Overview of capture distance (a), an aggregate, created without inclusion of a capture distance, (b), three possible random walks of a particle entering the highlighted section of (a) without a capture distance, (c), three possible random walks of a particle entering the highlighted section of (a) with a capture distance. The initial particle position is depicted in blue and final positions in red.

In Fig. 1, an overview of the representation and effect of the concept of a capture distance is provided. To enhance the clarity of the image, a 2D cluster is displayed. Here, and in the remainder of this work, a particle undergoing the random walk will be denoted with *p*_W_, whereas we will use *p*_Agg_ for those that are part the aggregate. In Fig. 1a, an aggregate is displayed, that was produced when assuming no capture effect to be present. The highlighted section of Fig. 1a is displayed in Fig. 1b with 3 random walks. The position of a wandering particle (*p*_W_), is depicted by the blue particle, the lines representing paths the diffusing particle may take and the 3 corresponding locations the particle may end up in when no capture effects are present are shown in red. Fig. 1c displays the same highlighted section in the presence of a capture zone. It can be seen that parts of the structure became inaccessible as a result of the screening effect of this region. When giving wandering particles the same initial paths as displayed in Fig. 1b, the presence of an active pull in Fig. 1c leads them to end up in different places as their path overlaps with the capture region in an earlier state of the random walk. As a result of the capture effect, particles are on average caught earlier, leading to less dense structures. We will use *ℓ*_c_ to represent the capture distance, which is defined as per eqn (1). Here, *r*_c_ denotes the radius of the capture distance and *r*_p_ is the particle radius. So *ℓ*_c_ = 1.0 in the absence of screening effects, like in Fig. 1b, whereas *ℓ*_c_ = 4.0 was used in Fig. 1c.1
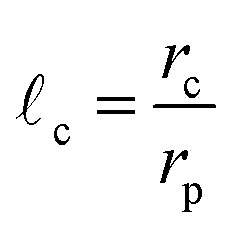


### 2.1. Capture distance

To compute the effective van der Waals forces, *F*_vdW_, of a cluster acting on a singular particle, we use the London-van der Waals equation for two identical spheres, eqn (2).^24^ Given the sensitivity of van der Waals forces with respect to particle distance, the closest particle within the cluster will induce the bulk of the pull. We can therefore simplify by only considering the forces between that particle, *p*_Agg_ and the wandering particle *p*_W_. In the ESI,† we perform sensitivity analyses to validate this assumption.2
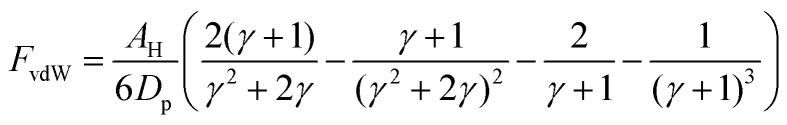
here, *A*_H_ is the Hamaker constant, *D*_p_ is the particle diameter and *γ* denotes the ratio of surface distance *d* between the wandering particle and the closest particle within the cluster to the particle diameter:3
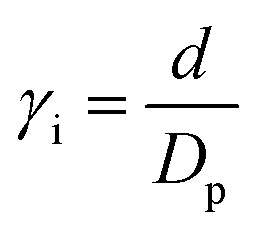


To find the location of the inter-particle capture distance, we use the fluctuation dissipation theorem,^25^ based on the Langevin equation, eqn (4).4
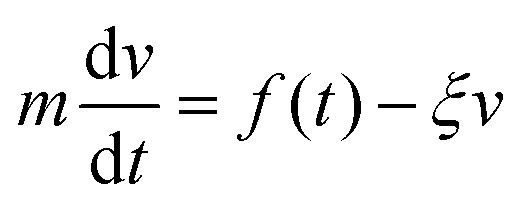
here, *m* is the mass of the singular particle, *t* is time, *f*(*t*) represents the fluctuating forces acting on the body, *ξ* is the friction factor and *v* is the particle velocity. Using eqn (4), the correlation time, after which the current velocity of a particle became uncorrelated with its initial velocity, can be found as:5
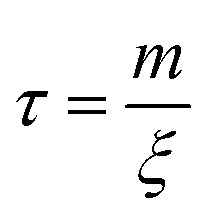
where6
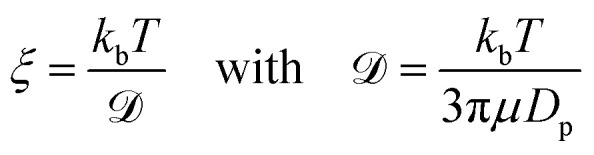
where *k*_b_ is the Boltzmann constant, *T* is the temperature and 𝒟 is the diffusion constant. To make this derivation leading from eqn (4) and (5), the assumption that 〈*f*(*t*)〉 = 0 has to be made. This assumption holds collectively for many particles, but is not necessarily true when considering a single particle at *t* < ∞.

To find the probability density of a Brownian particle in a constant drift, the Smoluchowski equation can be solved to find:7
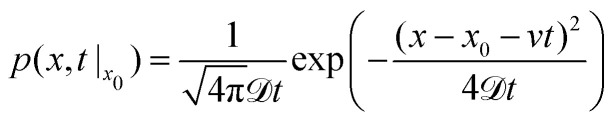


Finally, to solve eqn (7) a velocity, describing the drift, is required. In our case, this drift is induced by *F*_vdW_, which scales with surface distance, and therefore will change over time as the particle drifts towards or away from the cluster. To simplify, we assume *v* to remain constant within the correlation time, computed as per eqn (8). Here, we assume the effective *F*_vdW_ between the singular particle and cluster are equal to the force induced by the closest particle within the cluster. In the ESI† we perform a sensitivity analysis which validates this assumption.8
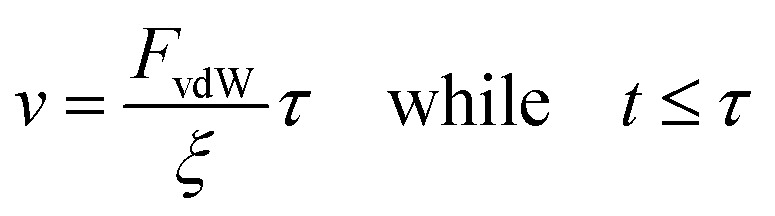


### 2.2. Model

Three types of aggregation dynamics are typically identified, reaction-limited, diffusion-limited and ballistic aggregation.^15,26^ In the reaction-limited regime, not all collisions lead to the particles sticking together. This can be modelled by introducing a sticking probability which is smaller than 1. Diffusion-limited and ballistic aggregation describe the path of a wandering particle by a random walk or a linear trajectory respectively. Collisions in these models do always result in aggregation.

As our work concerns the regime where primary particles are only just solidified and therefore still ‘sticky’, we consider our process to not be reaction limited. Furthermore, the particles considered in this study have such small volumes that they cannot build significant kinetic energy, needed to maintain a predictable direction of motion, therefore we model the system by using the diffusion-limited aggregation (DLA) approach.

A 3D off-grid DLA model, utilizing a random walk, was developed in Python to investigate our hypothesis. An initial, stationary, seed particle is introduced at (*x*,*y*,*z*) = (0,0,0) and new particles are introduced at a distance, scaling with current cluster size, and proceed with a random walk. New positions of wandering particles are computed using eqn (9). Here the displacement *Δ* is found by computing two random angles, *θ* and *ϕ* providing a direction to move in, and *δ*, the Euclidean distance between positions, which is kept constant.

9 (*x*,*y*,*z*)_*i*+1_ = (*x*,*y*,*z*)_*i*_ + *Δ*

with

10 *Δ* ∈ **Perm**(*δ* sin(*θ*)cos(*ϕ*), *δ* sin(*θ*)sin(*ϕ*), *δ* cos(*θ*))

The permutation performed by eqn (10) scrambles directions to eliminate biases that would otherwise be introduced if only randomizing the angles.

After every step, the Euclidean distance between the wandering particle and all particles within the cluster, *e*_*i*_, is computed to see whether *p*_W_ entered the capture region, *e*_*i*_ < *ℓ*_c_. Upon entering this zone, the collision point is set as the location of a particle within the cluster with the smallest surface distance to the current position of the random walker.

To speed up the process, additional random walkers, that do not interact with one another, are added in parallel, the total simultaneously running particles scaling with aggregate size. To prevent chain-reactions, where one collision leads to all nearby wandering particles colliding too, all walkers within 2 particle sizes of a colliding one are killed off. As in the work of Nicolás-Carlock *et al.*, we allow particles to wander away from the aggregate until twice the distance of their spawn point before removing them.

Fractal structures are typically described by eqn (11). Which, after rewriting, allows to find the fractal dimension, *D*_f_, of an aggregate with known size.11
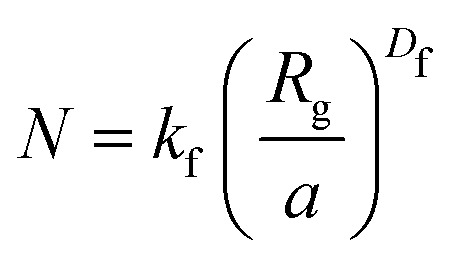


In eqn (11), *N* is the number of particles within the aggregate, *a* is the radius of the primary particles, *k*_f_ is the prefactor, assumed to be 1, and *R*_g_ is the effective radius of the gyration, which can be calculated as per eqn (12).12
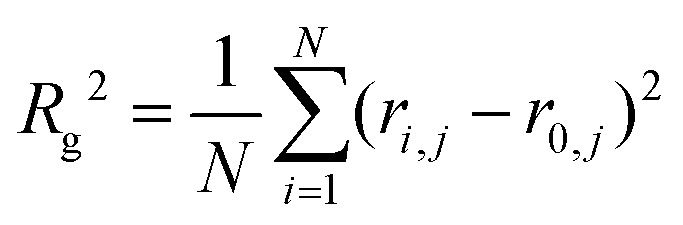
here *i* indicates the particle number within the aggregate, *j* represents the *x*, *y* or *z* coordinate, *r*_*i*,*j*_ is the position of the *i*^*th*^ particle and *r*_0,*j*_ is the position of the center of the cluster, calculated as follows:13
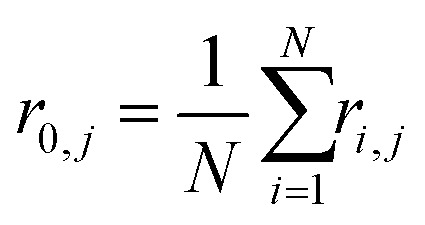


Since *R*_g_ is weighted, it is smaller than radii corresponding to the distance between the center of the cluster and the particle furthest removed from it, as one would determine from visual observation. Other works have not always explicitly mentioned how cluster radius was defined, but appeared to have used the approach analogous to visually observed radii (see ESI†). For an accurate assessment of *D*_f_, that method works best when the aggregate is fairly circular in a 2D, or spherical in a 3D system. Given a large enough cluster, this assumption may be valid. In our work however, we do not allow clusters to grow indefinitely, the variations within the clusters leading to inaccurate results using that approach, especially in 3D systems, which is why we opt to use eqn (12).

## 3. Results and discussion

### 3.1. Fractal dimension dependency

**Fig. 2:**
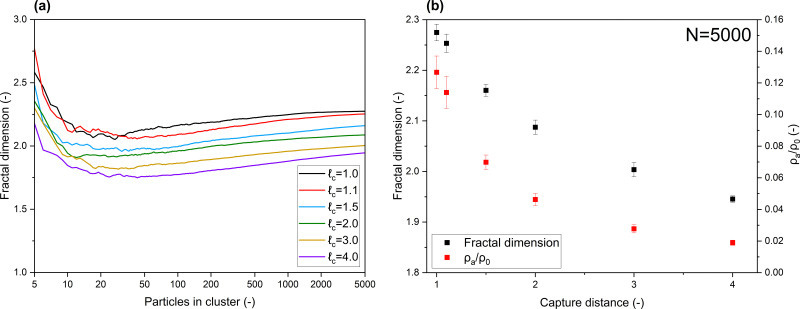
Effects of capture distance on properties of 3D aggregates, (a) development of fractal dimension of clusters produced with various capture distances, (b) obtained fractal dimensions and cluster densities of aggregates containing 5000 primary particles.

In Fig. 2, the properties of clusters produced with various capture distances are displayed. Fig. 2a demonstrates the development of the fractal dimension, note the log scale on the *x*-axis. A consistently lower *D*_f_ is obtained when clusters are produced with an increased capture distance. Final cluster diameters ranged from 79.1 (*ℓ*_c_ = 1.0) to 159.0 (*ℓ*_c_ = 4.0) primary particle sizes. With regard to typically sized nanoparticles (5–50 nm), this puts the produced clusters among the larger ones that are experimentally observed. Considering the slope of the curves in Fig. 2a, fractal dimensions of three dimensional clusters may indeed converge when building clusters with *N* → ∞, as shown to be the case for 2D clusters.^19^ On the scale in which the aggregates we are considering are typically found however, a pronounced difference in *D*_f_, induced by long-range interactions, is evident. Coefficients of variation within the data were found to be <1%, and can be found the ESI.†

The final fractal dimensions and densities of clusters containing 5000 primary particles are provided in Fig. 2b. Densities are normalized by the density of a primary particle. A clear decrease in cluster density is obtained when increasing the capture distance. Additionally, it can be observed that only minor variations are found among clusters created under the same conditions, highlighting the significance of our results. While less pronounced, the decreasing *D*_f_ with respect to capture distance is also found when using the approach used by Maekin, as demonstrated in the ESI.† Additionally, given that no such effect was obtained when using a 2D model, this underscores the limitations of studying screening effects in lower dimensions.

**Fig. 3:**
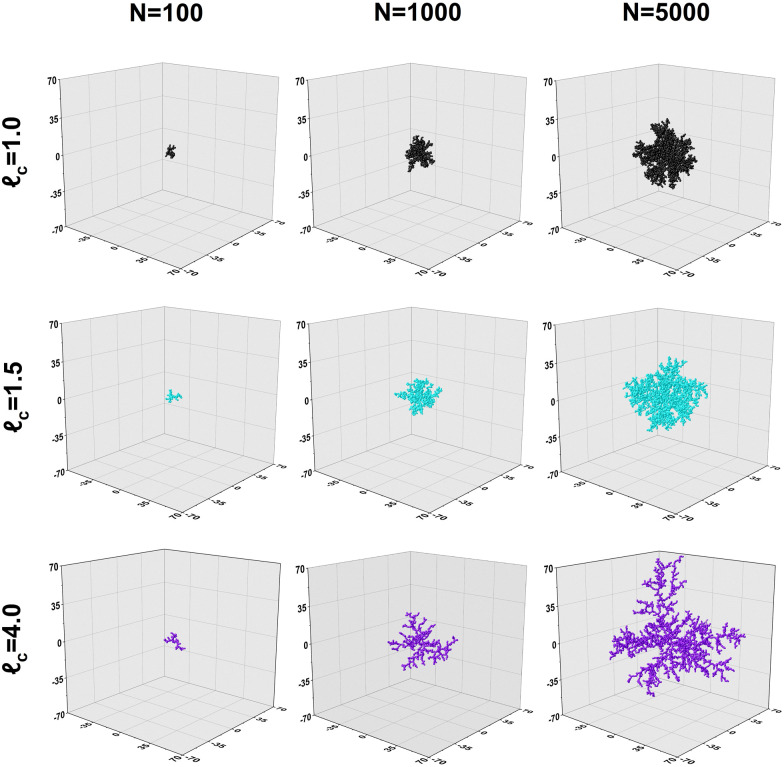
Development of aggregate structures produced with various capture distances.

Collectively, the subfigures in Fig. 2 demonstrate that, on the scale in which aggregates are typically found, increased capture distances lead to changes in the macro-properties of clusters as a result of more open structures being formed. A visualization of some produced clusters is provided in Fig. 3. The axes are normalized to the diameter of a primary particle. As indicated by the cluster properties presented in Fig. 2, it can be observed that increased capture distances lead to more open structures. It can be seen that our aggregates closely resemble those commonly observed by TEM. In particular, the 'net-like' structures, characteristic for nanoparticle aggregates are present in the model results as well.

### 3.2 Capture distance

**Fig. 4:**
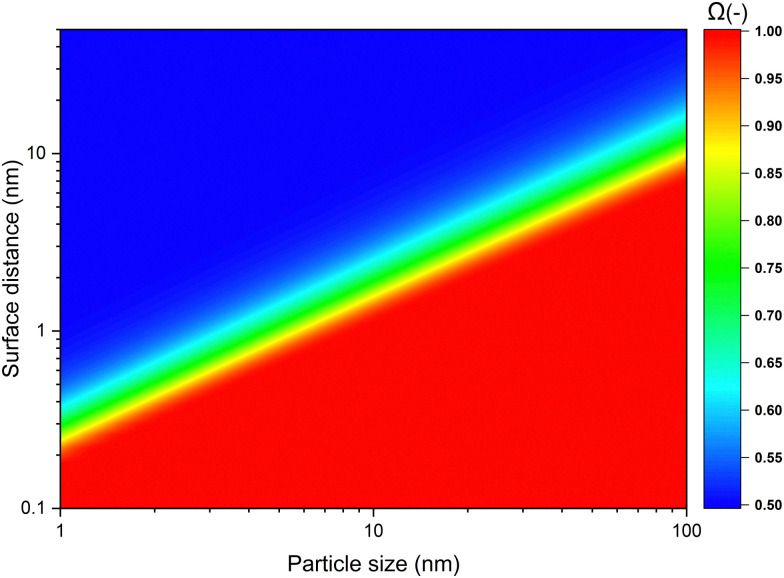
The probability of a particle moving towards another one as a result of van der Waals forces.

Next we determined the radius of the capture distance by using eqn (7) and material constants for SiO_2_, listed in Table 1. In Fig. 4, the probability of a singular particle moving towards another one as a function of particle size and initial surface distance, *Ω*, is shown. In the context of our work this represents the probability of a wandering particle *p*_W_ colliding with a particle within the aggregate *p*_Agg_. The region in deep blue represents the zone within which particle movement is completely stochastic, meaning the odds of the particle either approaching the cluster, or moving away from it are equal, hence the fraction of particles moving closer being 0.5. However, when the particle-cluster distance decreases, a transition region occurs, where the van der Waals force introduces a bias to the outcome of the stochastic Brownian motion. Finally, when surface distances are very small, particle movement is dominated by van der Waals forces, effectively rendering the process deterministic. Given the inevitability of a collision when particles reach beyond this capture distance, particle-cluster aggregation should be modelled by taking this region into account. It can be seen that the capture distance as determined by Fig. 4 for a 10 nm particle adds about 1.2 nm to its radius. The simplification of using only 1 particle to represent the forces exerted by the cluster was found to introduce an error of ≪5%, as shown in the ESI.† Given this result, we conclude that a realistic value of *ℓ*_c_ is about 1.1 for nanoparticles in the 5–100 nm range. While not as extreme as some of the capture distances we used for the modelling, based Fig. 2b we nevertheless expect a decrease of about 10% in cluster density due to the capture effect.

**Table 1:** Constants used to compute Fig. 4

Parameter	Value	Unit
*k*_b_	1.38 × 10^−23^	m^2^ kg s^−1^ K^−1^
*ρ*	2560	kg m^−3^
*T*	700	K
*μ*	3.45 × 10^−5^	kg m^−1^ s^−1^
*A*_H_	1.5 × 10^−19^	kg m^2^ s^−2^

Confirming our findings by direct experimental observation is very difficult. Characterization methods often require sample preparation that may alter the aggregate structure, in particular by compaction and, in the presence of liquid, by capillary forces. Values of *D*_f_ > 1.9 are typically reported when considering aggregates formed by sintering particles.^27^ However, reports using (small-echo) small-angle neutron scattering, which is less prone to alter aggregate features, indicate a wider range of fractal dimensions of ∼1.1–2.1^28–30^ for nanoparticle aggregates. While lower than the ∼2.3 predicted by a diffusion limited aggregation model in the absence of any capture effects, this is at least partially due to cluster-cluster aggregation. However, as these clusters are initially formed by particle-cluster interactions, a capture effect is still expected to contribute to even lower densities.

## 4. Conclusion

In this work, we assessed the effects of van der Waals interactions on the formation of nanoparticle aggregates. We hypothesized the existence of a critical distance between nanoparticles, beyond which collisions become inevitable as a result of attractive forces, affecting the aggregation process. Utilizing the Fluctuation Dissipation Theorem, we determined this region to extend about 10% beyond the particle radius for primary particles between 5–100 nm. Furthermore, we built a model to show that this effect alters the particle structure and results in a decrease in cluster density of ∼10%. Additionally, deviations between the results obtained from 2 and 3 dimensional models show that screening effects can not be accurately studied in by using 2D models. Experimental validation of our work is troublesome however, as it is unclear whether the predicted open structure is maintained upon further production and handling, especially in light of compaction. Furthermore, the complexity of accurately assessing structure on the aggregate scale, without affecting it, means very little experimental material is available for an accurate assessment.

While our work indicates capture effects to be present and significant, it relies on simplifications such as mono-sized spherical primary particles and a constant particle velocity within the correlation time. Furthermore, when determining the capture distance, we neglected all interactions besides van der Waals forces and Brownian motion. Additionally, used constants in this part of our work are subject to factors such as temperature and pressure, meaning that the outcome of this part of the work serves as an approximation. It would be interesting to see 3D models incorporating particle size distributions or allowing for different particle shapes. Furthermore, given that true systems are rarely fully ballistic or diffusion limited, the effect of combined particle dynamics in the context of screening effects would provide additional insight into real life systems.

## Data availability

Our script is available on GitHub and the 4TU data repository (open access), which also contains additional data on the produced aggregates for this work.

## Conflicts of interest

There are no conflicts to declare.

## Supplementary Material

CP-027-D4CP04235B-s001
