# Supplementary material for: On the structure of nanoparticle clusters: effects of long-range interactions
Source: Phys Chem Chem Phys. 2025 Jan 21;27(11):5476–81. doi: 10.1039/d4cp04235b (PMC11747963; doi:10.1039/d4cp04235b)
Supplement: CP-027-D4CP04235B-s001 [file CP-027-D4CP04235B-s001.pdf]

Supplementary Information (SI) for Physical Chemistry Chemical Physics.
This journal is © the Owner Societies 2025

SUPPLEMENTARY MATERIAL

On the structure of nanoparticle clusters: effects of long-range interactions

Rens Kamphorst*[a], Maximilian F. Theisen[a], Ankur D. Bordoloi[a], Samir Salameh[b], Gabrie M.H. Meesters[a], J. Ruud van Ommen*[a]

[a]*Department of Chemical Engineering, Delft University of Technology, Van der Maasweg 9, Delft, 2629HZ, the Netherlands*
[b]*FH Münster, Münster, Germany*

S1. Sensitivity of van der Waals interactions

S1.1. van der Waals forces

To compute the effective van der Waals forces, F_{vdW}, of a cluster acting on a singular particle, the London-van der Waals equation for two identical spheres was extended to include every particle within a cluster of N particles, shown in Eq. (S1).

$$F_{vdW} = \frac{A_H}{6D_p} \sum_{i=1}^{N} \left(\frac{2(\gamma_i + 1)}{\gamma_i^2 + 2\gamma_i} - \frac{\gamma_i + 1}{(\gamma_i^2 + 2\gamma_i)^2} - \frac{2}{\gamma_i + 1} - \frac{1}{(\gamma_i + 1)^3} \right) \tag{S1}$$

Here, A_H is the Hamaker constant, D_p is the particle diameter and γ_i denotes the ratio of surface distance d_i between the singular particle and particle i within the cluster to the particle diameter:

$$\gamma_i = \frac{d_i}{D_p} \tag{S2}$$

To compute d_i, we need to account for the position of each particle in the cluster, relative to the singular particle. To simplify, we consider linear strings of particles to represent the closest particles in a fractal structure. For the purposes of establishing the effective van der Waals forces between a single particle and a cluster, we consider two extreme cases, as demonstrated

in Fig. S1. The distance between particle A and the closest particle within the linear cluster (#1) is the same as the distance between particle B and the closest particle in the cluster (#3), however the distances between the other particles all differ, making the collective force acting on particle B larger.

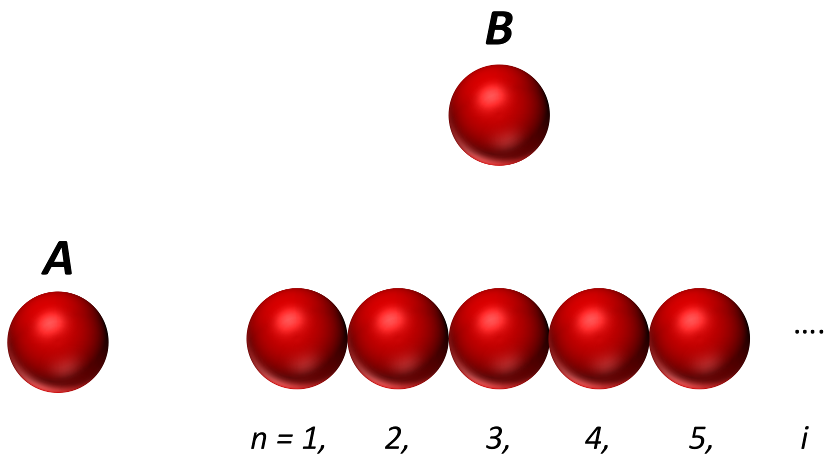

Figure S1: Particle-cluster configurations as considered for predicting effective van der Waals forces.

For the extreme cases considered in this study, d_i is computed as per Eq. (S3).

$$d_i = \begin{cases} d_0 + D_p(i-1), \text{if parallel} \\ \\ \sqrt{d_0^2 + \left(D_p \frac{i-\lambda}{2}\right)^2}, \text{if perpendicular} \end{cases} \tag{S3}$$

Here, d_0 denotes the surface distance to the singular particle and the closest particle in the cluster and λ takes values of 0 or 1 for clusters consisting of even and odd numbers of particles, respectively. Note that the computed surface distances for perpendicular clusters is only valid for clusters containing an odd number of particles and the singular particle is always located in the middle of the cluster, slightly overestimating the effective van der Waals force. Finally, we need to account for the angle at which individual particles within the cluster pull on the singular particle. Since we always consider

the singular particle to be located at the center of the string, the sideways components of the forces cancel out and the resulting force can be computed by multiplying Eq. (S1) by the correction factor α_i, where, as per Eq. (S4).

$$\alpha_i = cos\left(tan^{-1}\left(D_p \frac{i-\lambda}{2d_0}\right)\right) \tag{S4}$$

S1.2. Results

Computing the forces acting on a singular particle by all particles within a cluster comes at a computational cost. Therefore, before we determine the caption distance in real life aggregation processes, we assess the need to do so. From Eq. (S1) it is evident that van der Waals forces scale with both current cluster size and surface distance between each particle within the cluster and the single particle. However, its extreme sensitivity with respect to surface distance may result in the effect of the particle within the cluster with the smallest surface distance to outweigh even the collective effect of all others. This can easily be shown to be true in extreme cases, as the ratio between the surface distance of the closest particle in the cluster and any other particle, Q, can be computed as:

$$Q = \frac{r_i}{r_0} = \frac{r_0 + x_i}{r_0} \tag{S5}$$

Where r_0 and r_i are the distances to the closest particle and particle i respectively and x_i describes the absolute difference between them. Since $x_i > 0$, it is obvious that $\lim_{r_0 \to 0} Q = \infty$, meaning only the closest particle within the cluster is relevant upon close proximity. Furthermore, at $\lim_{r_0 \to \infty} Q = 1$, so at large distances, all particles become relevant. As demonstrated in Section S1.2 and Section S1.2, this is indeed the case for both parallel and perpendicular configurations. For these figures the normalized vdW forces are defined as the force acting on a cluster of a given number of particles divided by the force exerted by the closest particle within the cluster:

$$F_{vdW,norm} = \frac{\sum_i F_{vdW,n=i}}{F_{vdW,n=1}} \tag{S6}$$

The solid black line indicates the combination of surface distance and particles within the cluster where the force induced by the entire cluster exceeds the one induced by the closest particle within it by 5%. Collectively,

Section S1.2 and Section S1.2 demonstrate that multiple particles within a cluster should only be be taken into account when calculating the effective van der Waals forces on between a singular particle and a cluster when the cluster consists of many particles or when the distance between cluster and particle is $\gg 1\,\mathrm{nm}$.

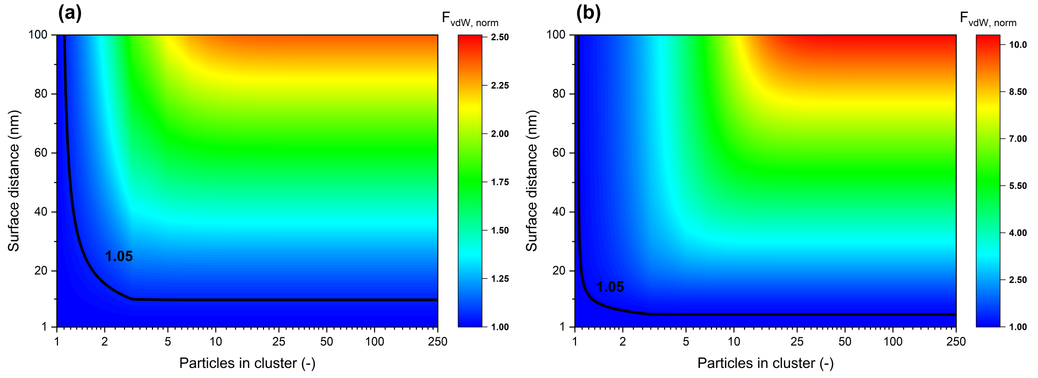

Figure S2: Normalized total forces acting between a singular particle and clusters of various surface distances and orientations: **(a)** Normalized van der Waals forces as a function of the number of particles in a cluster in parallel orientation and the surface distance between the closest particles within the cluster and a single particle, **(b)** Normalized van der Waals forces as a function of the number of particles in a cluster in perpendicular orientation and the surface distance between the closest particles within the cluster and a single particle, primary particle size = $10\,\mathrm{nm}$, $A_H = 1.5 \cdot 10^{-19}\mathrm{J}$

S2. 2D model

To validate our model, we adjusted it to produce 2D aggregates, which allowed for comparison of the output with previously published works. In Fig. S3 and Fig. S4 2D aggregates consisting of 1000 particles are displayed and compared to those of prior publications. It is evident that our output fully aligns with the one from the prior model, deviations arising from the stocastic nature of the aggregation process itself. Furthermore, Fig. S5 demonstrates the same results are obtained when computing the fractal dimension using the approach by Meakin in the original work.

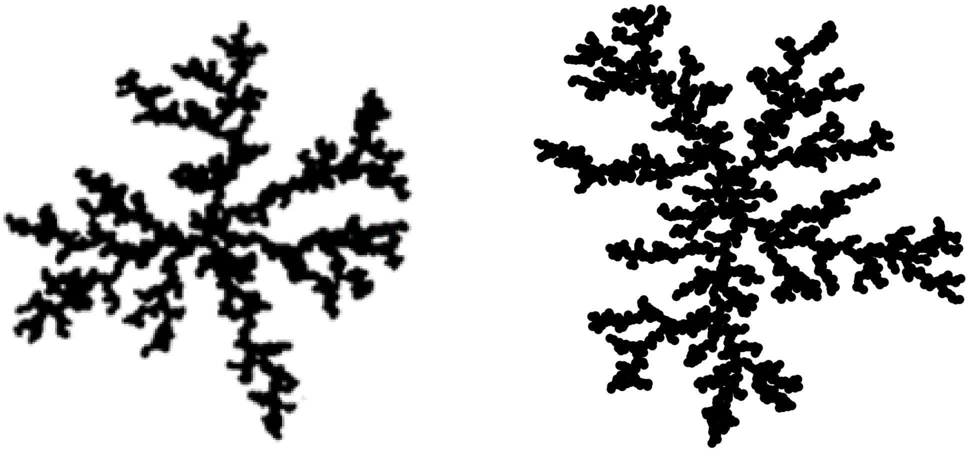

Figure S3: 2D aggregates containing 1000 particles produced from by Maekin (left) and our model (right) with a caption distance of ℓ_c =1.0

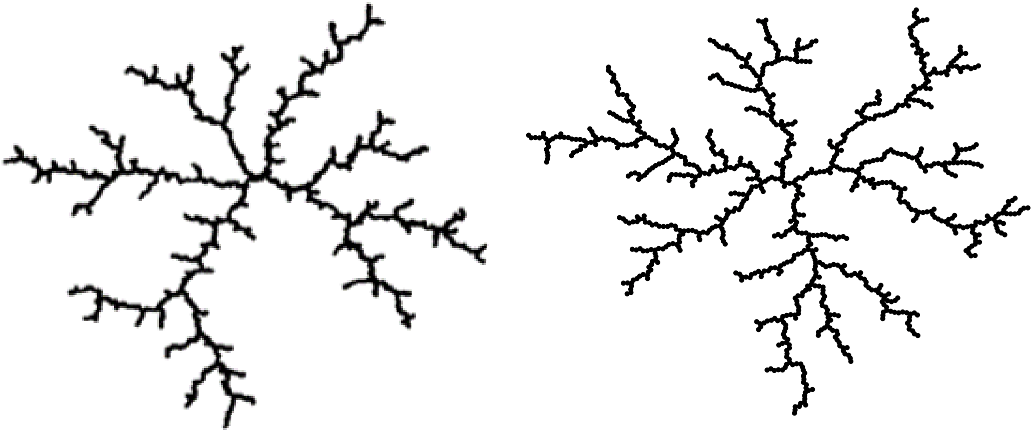

Figure S4: 2D aggregates containing 1000 particles produced from by Maekin (left) and our model (right) with a caption distance of ℓ_c =4.0

S3. Additional 3D model output

For the 3D model, we also used the method from prior works to confirm our finding to not be an artifact of the method to find D_f. In Fig. S6, the

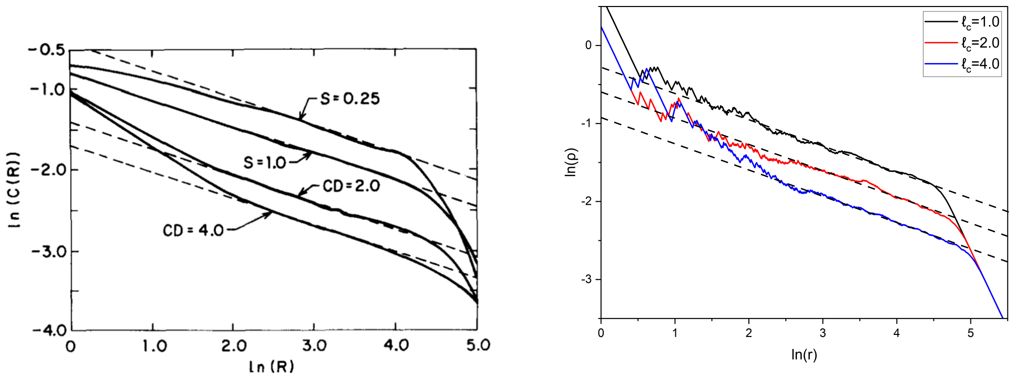

Figure S5: Density-radius correlation functions for various caption distances, obtained by 2D models. Left: Maekin, right: our model. Aggregates consisted of 5000-8000 primary particles. The dashed lines in both figures have a slope of -1/3

resulting dependencies of density and cluster size are provided. Here it can be seen that the slopes of curves obtained in 3D structures are indeed not parallel on the scale we consider. However, as we argue in the manuscript, this method is somewhat subjective, especially for smaller structures, where a fully linear zone is not obvious.

In Fig. S7, the coefficients of variation corresponding to Fig. 2a in the manuscript are provided. For the clusters of 5000 particles considered in the main text, it can be seen that none of the errors exceed 1 %, as indicated by the dashed line.

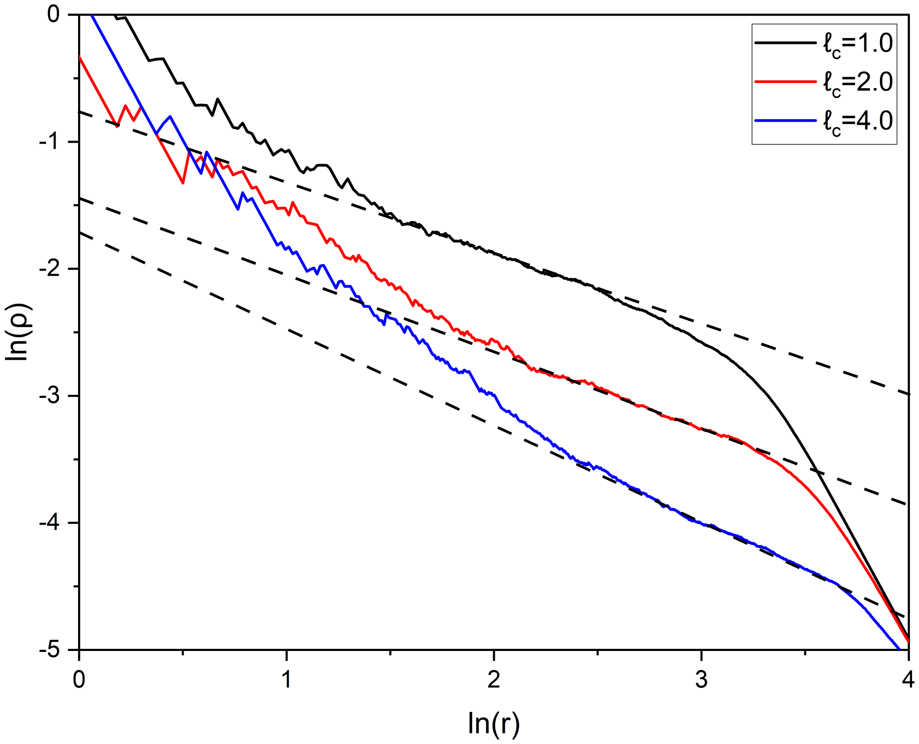

Figure S6: Density-radius correlation functions of 3D clusters, produced at various caption distances

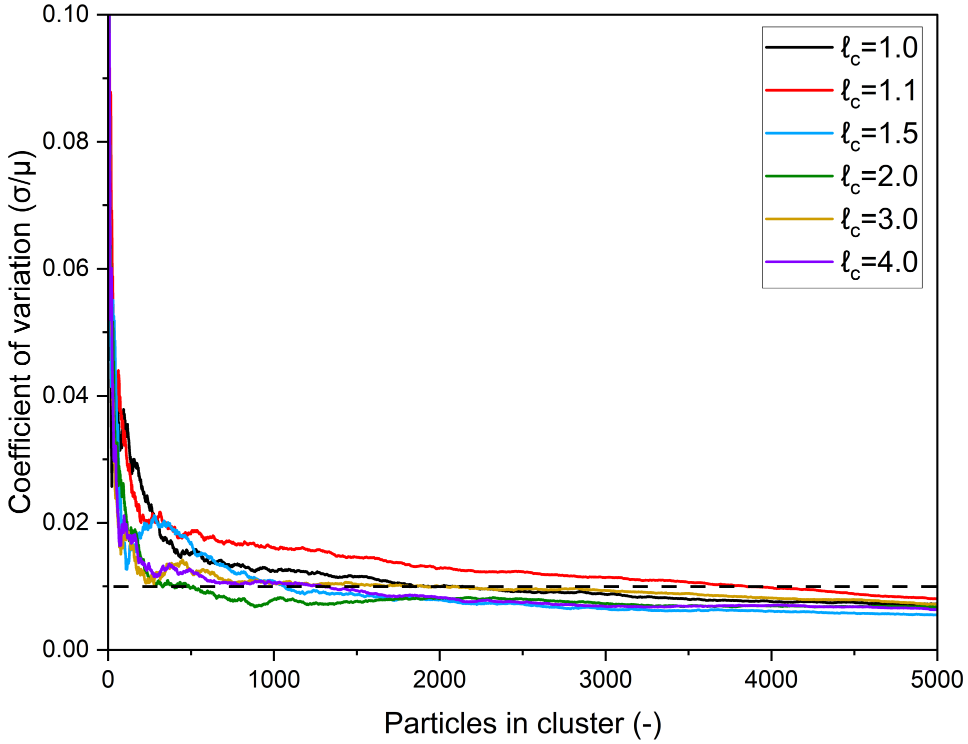

Figure S7: Coefficients of variation corresponding to sets of clusters produced under identical conditions. The horizontal dashed line indicates a value of 0.01
